# Predicting pathological highly invasive lung cancer from preoperative [^18^F]FDG PET/CT with multiple machine learning models

**DOI:** 10.1007/s00259-022-06038-7

**Published:** 2022-11-17

**Authors:** Yuki Onozato, Takekazu Iwata, Yasufumi Uematsu, Daiki Shimizu, Takayoshi Yamamoto, Yukiko Matsui, Kazuyuki Ogawa, Junpei Kuyama, Yuichi Sakairi, Eiryo Kawakami, Toshihiko Iizasa, Ichiro Yoshino

**Affiliations:** 1 Division of Thoracic Surgery, Chiba Cancer Centre, 666-2, Nitona-Cho, Chuo-Ku, Chiba, 260-8717 Japan; 2 Division of Nuclear Medicine, Chiba Cancer Centre, 666-2, Nitona-Cho, Chuo-Ku, Chiba, 260-8717 Japan; 3 Department of General Thoracic Surgery, Chiba University Graduate School of Medicine, Chiba, Japan; 4 Department of Artificial Intelligence Medicine, Chiba University Graduate School of Medicine, Chiba, Japan

**Keywords:** Positron emission tomography/computed tomography, Lung cancer, Radiomics, Surgery, Machine learning

## Abstract

**Purpose:**

The efficacy of sublobar resection of primary lung cancer have been proven in recent years. However, sublobar resection for highly invasive lung cancer increases local recurrence. We developed and validated multiple machine learning models predicting pathological invasiveness of lung cancer based on preoperative [^18^F]fluorodeoxyglucose (FDG) positron emission tomography (PET) and computed tomography (CT) radiomic features.

**Methods:**

Overall, 873 patients who underwent lobectomy or segmentectomy for primary lung cancer were enrolled. Radiomics features were extracted from preoperative PET/CT images with the *PyRadiomics* package. Seven machine learning models and an ensemble of all models (ENS) were evaluated after 100 iterations. In addition, the probability of highly invasive lung cancer was calculated in a nested cross-validation to assess the calibration plot and clinical usefulness and to compare to consolidation tumour ratio (CTR) on CT images, one of the generally used diagnostic criteria.

**Results:**

In the training set, when PET and CT features were combined, all models achieved an area under the curve (AUC) of ≥ 0.880. In the test set, ENS showed the highest mean AUC of 0.880 and smallest standard deviation of 0.0165, and when the cutoff was 0.5, accuracy of 0.804, F1 of 0.851, precision of 0.821, and recall of 0.885. In the nested cross-validation, the AUC of 0.882 (95% CI: 0.860–0.905) showed a high discriminative ability, and the calibration plot indicated consistency with a Brier score of 0.131. A decision curve analysis showed that the ENS was valid with a threshold probability ranging from 3 to 98%. Accuracy showed an improvement of more than 8% over the CTR.

**Conclusion:**

The machine learning model based on preoperative [^18^F]FDG PET/CT images was able to predict pathological highly invasive lung cancer with high discriminative ability and stability. The calibration plot showed good consistency, suggesting its usefulness in quantitative risk assessment.

**Supplementary Information:**

The online version contains supplementary material available at 10.1007/s00259-022-06038-7.

## Introduction

Lung cancer has one of the highest mortalities among cancer worldwide [1], and the 5-year survival rate is less than 50% for locally advanced lung cancer with invasion of other organs or mediastinal lymph node metastasis. In contrast, the 5-year survival rate is more than 80% for stage I lung cancer, for which surgery is the initial therapy, and a long-term survival can be expected with appropriate staging and treatment [2]. However, even stage I lung cancer is not uniformly pathologically invasive, and there are some high-grade lung cancers. Small-cell lung cancer [3] and large-cell neuroendocrine carcinoma [4] have a poor prognosis, as do adenocarcinoma with micropapillary component [5]. Recurrence rates and prognoses vary, and it is necessary to provide appropriate medical treatment for each patient rather than uniform local therapy and postoperative follow-up. In terms of surgery, lobectomy is the standard procedure for primary lung cancer, but the indication of sublobar resection for small lung cancer has been verified in the large-scale clinical trials JCOG0802/WJOG4607L [6] and CALGB140503 [7]. The underlying reason is that sublobar resection is expected to be non-inferior to lobectomy with respect to overall survival in small lung cancers. JCOG0802/WJOG4607L showed that local recurrence was more likely with sublobar resection than lobectomy, but the overall survival was superior due to an improvement in deaths from other diseases [6].

However, radical treatment for recurrence may require a pneumonectomy, and the burden of radiation therapy and chemotherapy is large. Therefore, the selection of procedure should depend on the risk of recurrence. Lung cancers prone to recurrence tend to have lymphovascular invasion or pleural invasion, and an increase in local recurrence has been reported in cases in which sublobar resection was performed for highly invasive cancer [8]. Koike et al. reported that lymphatic invasion and pleural invasion were independent predictors of local recurrence in sublobar resection, with hazard ratios of 3.824 and 2.272, respectively. However, the preoperative diagnosis of invasiveness is still difficult in the clinical setting since the pathological invasiveness of lung cancer is evaluable only after scrutinizing the pathological specimen. Therefore, we explored methods for estimating the presence of invasion based solely on preoperative radiomic features.

The invasiveness of lung cancer has been conventionally evaluated based on tumour diameter and consolidation tumour ratio (CTR) on computed tomography (CT) images [9]. Although higher CTR tends to indicate higher malignancy, CT findings do not always match the pathologic invasive findings in individual cases [10]. In addition, many lung cancers are solid nodules without ground-glass opacity, and false-positive results occur frequently with CTR [11]. Other than CT scan, fluorodeoxyglucose-positron emission tomography (FDG-PET) is recommended for evaluating the presence of lymph node metastases and inspecting distant metastases [12]. FDG-PET is also useful for a qualitative evaluation reflecting the tumour metabolism. Not only the maximum standardised uptake value (SUV_max_) but also the metabolic tumour volume (MTV) and total lesion glycolysis (TLG) are useful for making a diagnosis, evaluating the curative effect, and determining the prognosis [13, 14]. Furthermore, with the recent development of radiomics, which aims to extract quantitative radiological features from medical images in a high-throughput manner for diagnostic and therapeutic applications, medical imaging modalities including FDG-PET are expected to fill new clinical roles.

The application of machine learning to radiomics has been shown to improve the predictive performance in the diagnosis and prognosis prediction [15]. Many machine learning models with CT images have been reported in lung cancer [16, 17], but few analyses have been performed on FDG-PET images, and no studies have predicted highly invasive lung cancer restricted to patients who had undergone surgery. Even in the reported PET image studies, the small sample size has limited the evaluation of the prediction models [18].

In the present study, we constructed and validated a PET/CT radiomics-based machine learning model to predict pathological highly invasive lung cancer in a large cohort of patients who had undergone surgery for lung cancer. This study further analysed the predictive performance and its application to clinical practice, and compared machine learning model to the CTR on CT images. We also evaluated the performance when the histological type was limited to adenocarcinoma (Adc) or squamous cell carcinoma (Sqc) and when the tumour diameter was limited to ≤ 3 cm or ≤ 2 cm.

## Materials and methods

### Patient selection

**Fig. 1:**
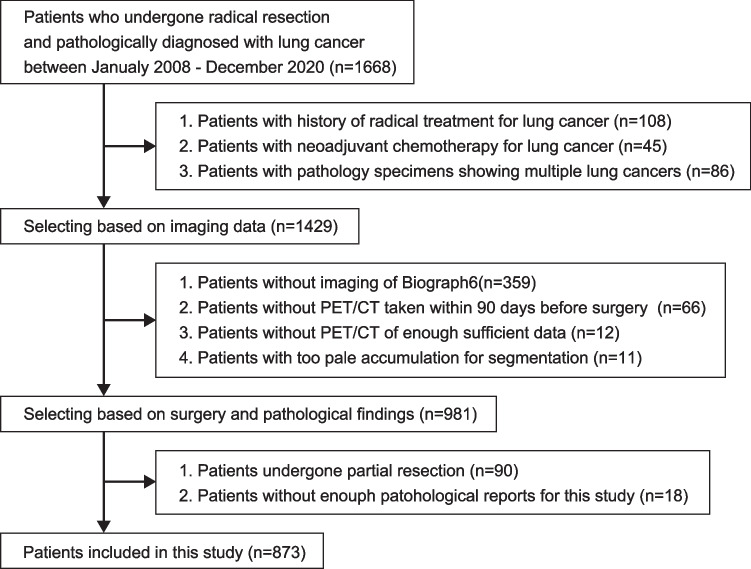
The flow chart of patient selection. A total of 1668 patients underwent lung cancer resection during the study period. Ultimately, 873 patients met the criteria and were included in the study

This study is a retrospective, single-centre study. Patients who underwent surgery for primary lung cancer between January 2008 and December 2020 at the Department of Thoracic Surgery, Chiba Cancer Centre, were included. Exclusion criteria were as follows: (i) a history of radical treatment for lung cancer; (ii) neoadjuvant chemotherapy for lung cancer; (iii) patients with pathology specimens showing multiple lung cancers; (iv) no [^18^F]FDG PET/CT using a Siemens Biograph 6 LSO (Siemens, Erlangen, Germany) within 3 months before surgery; (v) missing analysable imaging data; (vi) weakly integrated and not suitable for an analysis; (vii) partial resection performed; and (viii) pathology report not sufficient to diagnose pathological highly invasive lung cancer. Cases of partial resection were excluded because the lymph nodes were not evaluated. A flowchart of the selection criteria is shown in Fig. 1. Ultimately, 873 of 1668 patients met the criteria and were eligible. This study was approved by the Ethics Review Committee at our institution (No. R04-134), and written informed consent was waived for this retrospective study.

### Pathological findings

Pathological highly invasive lung cancer was defined in cases with any of the following: (i) lymph node metastasis; (ii) vascular invasion; (iii) lymphatic invasion; (iv) pleural invasion; or (v) intrapulmonary metastasis. The control group was defined as cases of lung cancer without any of the above findings.

### Image acquisition

[^18^F]FDG PET/CT images were obtained using a Biograph 6 PET/CT scanner (Siemens Healthcare) at the Chiba Cancer Centre. The median number of days between the date of PET/CT and the date of surgery was 34 (interquartile range: 23–45). Imaging was performed following the guidelines of the Japanese Society of Nuclear Medicine. [^18^F]FDG at 4 MBq/kg of body weight was injected before image acquisition, and imaging started 60 min after the acquisition. PET was performed for 2 min per bed, with a shorter imaging time per bed for higher doses and longer imaging time for lower doses to reduce the effect of dose differences. The CT slice thickness was 5 mm. The point spread function algorithm corrected the image reconstruction.

### Tumour segmentation

PET and CT images were retrieved from the electronic medical record, loaded by 3D slicer software, version 4.11, and used for lung cancer segmentation. *Grow from seeds* implemented in 3D slicer was used to segment lung cancer in CT images. *Grow from seeds* achieves segmentation by starting from pixels that are simply pointed out as lung cancer and background pixels and enlarging the region of interest [19]. *PET Tumor Segmentation*, a semi-automatic method, was used for PET image segmentation. *PET Tumor Segmentation*, which is faster and more consistent than manual segmentation [20], can be implemented as an extension of 3D slicer. The SUV_max_, SUV_mean_, MTV, and TLG of the segmented regions were calculated using the *PET-IndiC*, an extension of 3D slicer.

### Radiomics feature extraction

The Python package *Pyradiomics* (version 3.0.1 https://github.com/Radiomics/pyradiomics) was used for feature extraction. Both PET and CT images were resampled to a uniform voxel size of 2.0 × 2.0 × 2.0 by B Spline completion. Discretization of PET and CT images was set to a bin width of 0.5 for PET images and 25 for CT images [21]. A total of 3190 features (1595 each) were extracted. The original 107 features were calculated for each PET and CT image. One thousand four hundred eighty-eight features were calculated with filters of square, square root, logarithm, exponential, logarithm, wavelet, and Laplacian of Gaussian, and three sigma values of 2.0, 3.0, and 4.0 were used in the Laplacian of Gaussian filter.

### Model development

The workflow of this study consists of two steps (Supplementary Fig. 1). The first step involves analysing only CT, only PET, and combined PET/CT features (hereafter denoted as PET/CT) to evaluate prediction performance and stability of each machine learning model. The patient cohort was divided 70% into a training set and 30% into a test set, and feature standardization and selection were performed based on the training set and applied to the test set. Feature selection was performed with Boruta [22], a feature selection method based on the variable importance of Random Forest (RF). Boruta selects variables by creating random shadow variables and repeatedly comparing variable importance to them. Although several feature selection methods exist, Boruta has been validated in many studies, and Degenhardt et al. concluded that Boruta was more effective than other methods in selecting a small subset containing the best predictor variables in omics data [23]. In the present study, the parameters of Boruta were set to n_estimators 300, perk 100, and alpha 0.05.

The machine learning models used in our study were logistic regression (LR), support vector machine (SVM), K-nearest neighbour (KNN), RF, light gradient boosting machine (LGB) [24], deep neural net (DNN), and TabNet [25]. As deep neural models, not only DNN but also TabNet, which is specialised for tabular data, were used. We also established the ensemble model (ENS), which averaged the prediction probabilities of all models in the test set. For machine learning models other than TabNet, hyperparameters were optimised using fivefold cross-validation with area under the curve (AUC) as the evaluation metrics. TabNet was optimised using the validation set after pretraining with fixed parameters. Details of the hyperparameter settings are shown in Supplementary Table 1. The same procedure was repeated for 100 iterations with different random seeds to evaluate prediction performance and stability, from the division of patients to constructing the machine learning model as one iteration. The mean and standard deviation (SD) of the AUC, accuracy, F1, precision, and recall were calculated as evaluation metrics.

For the second step of the analysis, a calibration plot and decision curve analysis (DCA) [26] based on predicted probability were performed for clinical use. A calibration plot visualises the reliability of the predicted probability comparing the probability and actual proportions. A DCA uses the theoretical relationship between the relative harms of false positives and false negatives to indicate the range or amount of benefit from changing the thresholds on which treatment selection is based. Net Benefit of a model is given by:

$$\mathrm{Net}\;\mathrm{Benefit}=\frac{True\;Positive\;Count}n-\frac{False\;Positive\;Count}n(\frac{p_t}{1-p_t})$$

Here, ${p}_{t}$ is the changing threshold probability. The higher the net benefit, the more beneficial the model. To calculate the probability for all patients, all models except for TabNet were analysed by nested fivefold cross-validation with the inner loop of fivefold cross-validation. TabNet was analysed with the inner loop of hold-out. In addition, we performed an analysis limited to Adc, Sqc, and tumours with a horizontal section diameter ≤ 3 cm and ≤ 2 cm, based on the predicted probabilities calculated in the analysis of all patients. In addition, a comparison of the CTR was also performed. The CTR was measured based on thin-slice CT if taken within 3 months before surgery.

### Statistical analyses

As appropriate, categorical or continuous variables were compared with Fisher’s exact test, *t*-tests, or the Mann–Whitney* U* test. All analyses were two-tailed, with *P* < 0.05 indicating a significant difference. Statistical analyses of the patient background and a DCA were performed using the R software program (version 3.6.3, http://www.R-project.org). Python (version 3.7) with the scikit-learn package (version 1.0.2) and Pytorch (version 1.10.2) were used to build machine learning models and evaluate their predictive performance.

## Results

### Patient characteristics

A total of 873 lung cancer patients were ultimately eligible. Pathologically 317 patients were in the control group without any invasion findings, and 556 had highly invasive lung cancer. Patient characteristics are shown in Table 1.

**Table 1:** Patient characteristics

Total (*n* = 873)	Control group (*n* = 317)	Highly invasive (*n* = 556)	*P* value
Age, mean ± SD	69.1 ± 8.4	68.7 ± 9.2	0.49
Sex			< 0.001
Female	141 (44.5%)	137 (24.6%)	
Male	176 (55.5%)	419 (75.4%)	
Pack years, median (IQR)	16 (0–40)	40 (15–57)	< 0.001
CT tumour size, median (IQR)	24.7 (20.1 – 31.6)	35.0 (26.0 – 47.4)	< 0.001
FDG-PET findings, median (IQR)			
SUVmax	2.99 (1.58—6.08)	13.4 (7.75 – 19.22)	< 0.001
SUVmean	1.85 (1.23—2.72)	4.84 (3.25 – 6.91)	< 0.001
MTV	3.02 (1.78 – 6.29)	12.1 (5.15 – 29.00)	< 0.001
TLG (SUVmean*Volume)	5.99 (2.43—16.42)	62.4 (19.35 – 188.24)	< 0.001
Surgical procedure			< 0.001
Lobectomy	245 (77.3%)	523 (94.1%)	
Segmentectomy	72 (22.7%)	33 (5.9%)	
Tumor histology			< 0.001
Adenocarcinoma	272 (85.8%)	324 (58.3%)	
Squamous cell carcinoma	37 (11.7%)	154 (27.7%)	
Others	8 (2.5%)	78 (14.0%)	
Pathological stage			-
1A	265 (83.6%)	96 (17.3%)	
1B	41 (12.9%)	200 (36.0%)	
2A	10 (3.2%)	81 (14.6%)	
2B	1 (0.3%)	29 (5.2%)	
3A	0	110 (19.8%)	
3B	0	6 (1.1%)	
4	0	34 (6.1%)	
Pathological findings (%)			-
Vascular invasion	0	458 (82.3%)	-
Lymphatic invasion	0	253 (45.5%)	-
Pleural invasion	0	307 (55.2%)	-
Pulmonary metastasis	0	34 (6.1%)	-
Lymph node metastasis	0	213 (38.3%)	-

The patients’ background characteristics are presented. Categorical variables were analysed with Fisher’s exact test and continuous variables with a *t*-test or the Mann–Whitney *U* test. *CT*, computed tomography; *FDG-PET*, fluorodeoxyglucose positron emission tomography; *SUV*, standardised uptake value; *MTV*, metabolic tumour volume; *TLG*, total lesion glycolysis

Age was not significantly different between the control group and highly invasive group. The highly invasive group showed higher proportions of male subjects (*P* < 0.001) and smokers (*P* < 0.001) than the control group. The tumour diameter on CT was significantly larger in the highly invasive group than in the control group (*P* < 0.001). Furthermore, according to FDG-PET images, the SUV_max_ (*P* < 0.001) and the SUV_mean_ (*P* < 0.001) were higher and the MTV was larger (*P* < 0.001), resulting in a higher TLG (*P* < 0.001) in the highly invasive group than in the control group. Lobectomy was more frequently selected in the highly invasive group than in the control group (*P* < 0.001). In terms of histology, Adc was more common in the control group than in the highly invasive group. Vascular invasion was the most common reason for highly invasive lung cancer (82.3%), followed by lymphatic invasion in 45.5%, pleural invasion in 55.2%, pulmonary metastasis in 6.1%, and lymph node metastasis in 38.3% of cases.

### Machine learning prediction

Seven machine learning models and a simple average of predictions on the test set were established to evaluate prediction performance. The above steps were repeated 100 iterations to obtain the mean and SD of predicted probabilities. In the training set, the model was constructed with the AUC as the evaluation metrics (Supplementary Table 2). All models except for TabNet were cross-validated and achieved a mean AUC > 0.88 for PET/CT, > 0.87 for PET, and > 0.85 for CT. LGB achieved the highest mean AUC of 0.894 (SD 0.00838) for PET/CT and 0.885 (SD 0.00921) for PET. LGB and DNN achieved the highest AUC of 0.865 for CT and mean SDs of 0.00969 and 0.00982, respectively.

**Fig. 2:**
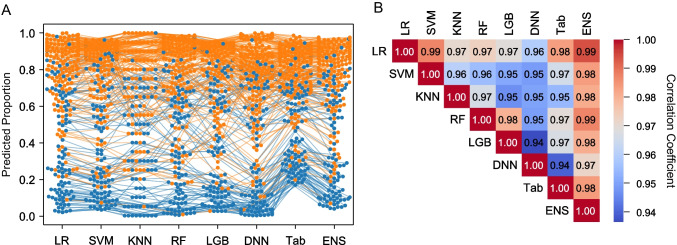
The predicted probability and coefficient correlation of all models. **A** The probability of each model in one test set is visualized in the swarm plot. Identical cases are connected by straight lines. Plots indicate highly invasive lung cancer (orange) and lung cancer in the control group (blue). **B** Correlation coefficients of probability for each model are shown

The results in the test set are shown in Table 2. For evaluations except for the AUC, the threshold was set to 0.5. For PET/CT, LR and ENS achieved the highest mean AUC of 0.880 and mean SDs of 0.0168 and 0.0165 respectively, outperforming SVM (AUC 0.867, SD 0.0191), KNN (AUC 0.868, SD 0.0167), RF (AUC 0.875, SD 0.0170), LGB (AUC 0.874, SD 0.0182), DNN (AUC 0.875, SD 0.0194), and TabNet (AUC 0.871, SD 0.0170). For PET alone, ENS achieved an AUC of 0.872 (SD 0.0177), the best performance. Although less predictive performance than PET/CT, all models performed well. For CT alone, ENS achieved an AUC of 0.856 (SD 0.0183), which showed similarly high predictive performance, but all models performed worse than PET alone or PET/CT. Figure 2A shows the probability of each model in one test set. Cases with high or low predictive probability tended to show equal probability in all models, while cases with probability around 0.5 were scattered with markedly different values in each model. Figure 2B shows the correlation coefficient of probability for each model. The ENS had a coefficient of 0.97 or higher for all models, indicating a strong correlation. Conversely, the deep model had a weak correlation with a coefficient of less than 0.96 for all models except for ENS.

**Table 2:** Performance of the machine learning models for the test set

	AUC (SD)	Accuracy (SD)	F1 score (SD)	Precision (SD)	Recall (SD)
CT					
LR	0.854 (0.0167)	0.792 (0.0330)	0.845 (0.0198)	0.806 (0.0473)	**0.892 (0.0383)**
SVM	0.842 (0.0206)	0.795 (0.0208)	0.838 (0.0182)	**0.837 (0.0262)**	0.842 (0.0330)
KNN	0.845 (0.0215)	0.791 (0.0196)	0.842 (0.0166)	0.809 (0.0265)	0.879 (0.0266)
RF	0.851 (0.0187)	0.798 (0.0194)	**0.847 (0.0161)**	0.817 (0.0249)	0.879 (0.0260)
LGB	0.848 (0.0202)	0.792 (0.0202)	0.842 (0.0161)	0.810 (0.0264)	0.878 (0.0250)
DNN	0.853 (0.0188)	0.794 (0.0207)	0.843 (0.0173)	0.818 (0.0312)	0.872 (0.0398)
TabNet	0.848 (0.0209)	0.794 (0.0202)	0.844 (0.0168)	0.812 (0.0363)	0.882 (0.0442)
ENS	**0.856 (0.0183)**	**0.798 (0.0188)**	0.846 (0.0158)	0.819 (0.0251)	0.876 (0.0281)
PET					
LR	0.869 (0.0184)	0.788 (0.0415)	0.841 (0.0236)	0.810 (0.0480)	0.878 (0.0342)
SVM	0.864 (0.0179)	0.792 (0.0207)	0.841 (0.0193)	0.815 (0.0330)	0.873 (0.0495)
KNN	0.859 (0.0201)	0.790 (0.0195)	0.840 (0.0173)	0.814 (0.0277)	0.868 (0.0284)
RF	0.867 (0.0193)	0.791 (0.0200)	0.839 (0.0169)	**0.821 (0.0288)**	0.860 (0.0283)
LGB	0.866 (0.0189)	0.786 (0.0200)	0.835 (0.0173)	0.817 (0.0273)	0.856 (0.0264)
DNN	0.869 (0.0181)	0.788 (0.0206)	0.836 (0.0197)	0.820 (0.0374)	0.857 (0.0532)
TabNet	0.866 (0.0177)	0.783 (0.0284)	0.839 (0.0185)	0.796 (0.0454)	**0.893 (0.0530)**
ENS	**0.872 (0.0177)**	**0.793 (0.0189)**	**0.842 (0.0160)**	0.817 (0.0280)	0.871 (0.0265)
PET/CT					
LR	0.880 (0.0168)	0.806 (0.0188)	0.852 (0.0151)	0.824 (0.0244)	0.883 (0.0215)
SVM	0.867 (0.0191)	**0.807 (0.0195)**	**0.853 (0.0158)**	**0.825 (0.0281)**	0.885 (0.0282)
KNN	0.868 (0.0167)	0.797 (0.0189)	0.847 (0.0150)	0.811 (0.0278)	**0.887 (0.0280)**
RF	0.875 (0.0170)	0.797 (0.0202)	0.844 (0.0167)	0.823 (0.0264)	0.869 (0.0290)
LGB	0.874 (0.0182)	0.797 (0.0201)	0.845 (0.0164)	0.820 (0.0243)	0.873 (0.0261)
DNN	0.875 (0.0194)	0.802 (0.0204)	0.848 (0.0178)	0.824 (0.0330)	0.877 (0.0456)
TabNet	0.871 (0.0170)	0.794 (0.0238)	0.845 (0.0173)	0.810 (0.0368)	0.887 (0.0406)
ENS	**0.880 (0.0165)**	0.804 (0.0185)	0.851 (0.0147)	0.821 (0.0249)	0.885 (0.0240)

The mean value and standard deviation of 100 times for each evaluation index are shown. Bold indicates the highest values. *CT*, computed tomography; *LR*, logistic regression; *SVM*, support vector machine; *KNN*, K-nearest neighbour; *RF*, random forest; *LGB*, light gradient boosting machine; *DNN*, deep neural net; *ENS*, ensemble; *PET*, positron emission tomography

### Calibration plot and DCA results

**Fig. 3:**
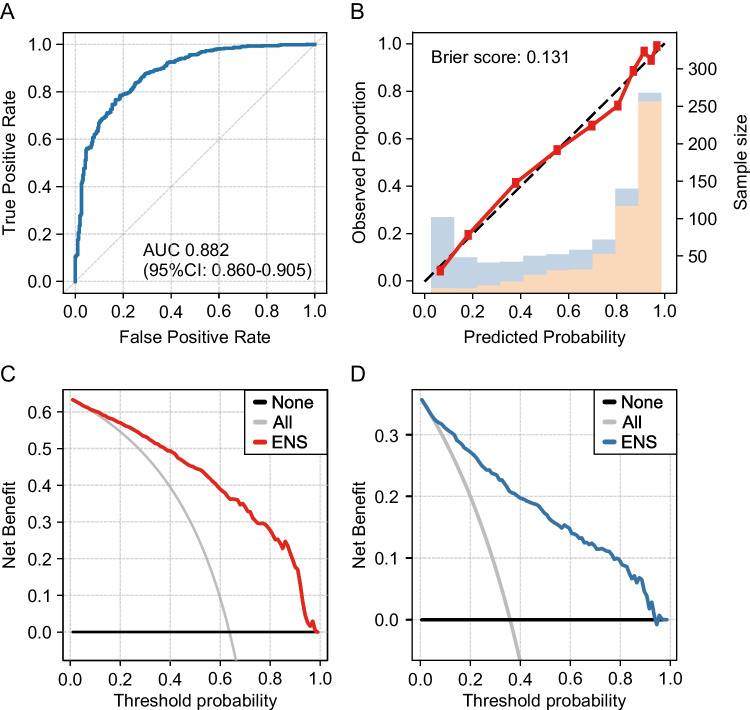
Machine learning model with ENS for all cases. **A** ROC curve for ENS model in differentiating pathological highly invasive and non-invasive lung cancer. **B** The calibration plot shows the consistency between the predicted probability of highly invasive cancer and the actual rate. Bars indicate the group with highly invasive lung cancer (orange) and the control group (blue) per interval of predicted probability. **C** A DCA for pathological highly invasive cancer. **D** A DCA for the control group

The probability of including in the highly invasive lung cancer was calculated for all lung cancers based on nested fivefold cross-validation. Figure 3 shows the analysis of ENS based on all cases, and the AUC was 0.882 (95% CI: 0.860–0.905). In the calibration plot, the predicted probability closely matched the actual highly invasive cancer probability, with a Brier score of 0.131 (Fig. 3B). In the decision curve, the net benefit was higher for using the probability of ENS than for the assumption that all lung cancers were highly invasive if the threshold probability was greater than 6% (Fig. 3C). For the control group, a threshold probability between 2 and 94% was valid (Fig. 3D).

**Fig. 4:**
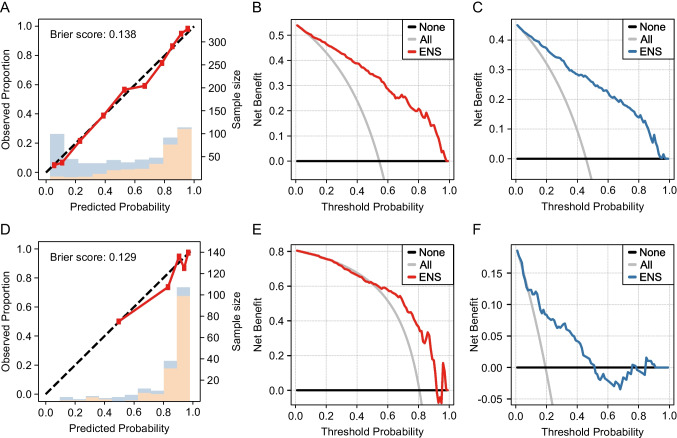
Machine learning model with ENS for adenocarcinoma or squamous cell carcinoma. **A**, **B**, **C** and **D**, **E**, **F** indicate data for adenocarcinoma and squamous cell carcinoma, respectively. **A**, **D** The calibration plot shows the group with highly invasive cancer (orange) and the control group (blue) per interval of the predicted probability. The predicted probability of highly invasive lung cancer and the actual proportion was consistent. **B**, **E** A DCA for pathological highly invasive lung cancer for adenocarcinoma and squamous cell carcinoma. **C**, **F** A DCA for the control group of adenocarcinoma and squamous cell carcinoma

**Fig. 5:**
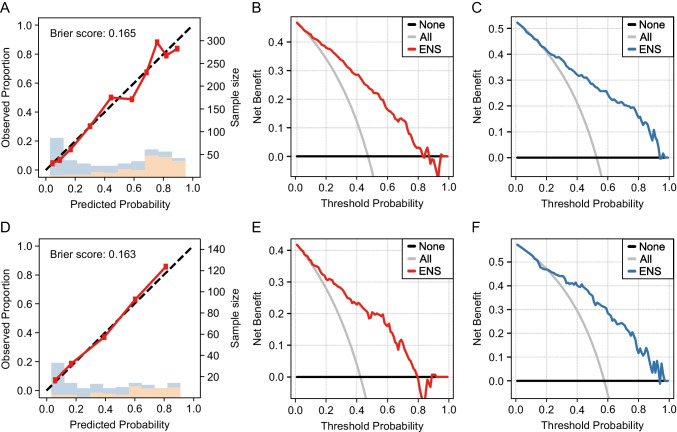
Machine learning model with ENS for tumours ≤ 3 cm or ≤ 2 cm in diameter. **A**, **B**, **C** and **D**, **E**, **F** indicate data for tumours ≤ 3 cm and ≤ 2 cm in diameter, respectively. **A**, **D** A calibration plot shows the consistency between the predicted probability of highly invasive cancer and the actual proportion. Bars indicate the group with highly invasive cancer (orange) and the control group (blue) for each interval of predicted probability. **B**, **E** A DCA for pathological highly invasive cancer for tumours ≤ 3 cm and ≤ 2 cm in diameter. **C**, **F** A DCA for control group of tumors ≤ 3 cm and ≤ 2 cm in diameter

Next, patients with Adc (Fig. 4A, B, C) and Sqc (Fig. 4D, E, F) were extracted from the results based on all patients. The receiver operating curves (ROCs) are shown in Supplementary Fig. 2. For Adc, ENS achieved an AUC of 0.885 (95% CI: 0.859–0.911) and a Brier score of 0.138. The decision curve showed validity with a predictive probability threshold of 3–98% for highly invasive lung cancer and 2–97% for the control group. For Sqc, the AUC was 0.789 (95% CI: 0.704–0.875), and the Brier score was 0.129. The decision curve showed validity with a predictive probability threshold of 49–91% for highly invasive lung cancer and 9–51% for the control group. For tumours with a diameter of ≤ 3 cm (Fig. 5A, B, C), ENS achieved an AUC of 0.836 (95% CI: 0.798–0.874) and Brier score of 0.165, and for tumours with a diameter of ≤ 2 cm (Fig. 5D, E, F), ENS achieved an AUC of 0.834 (95% CI: 0.765–0.903) and Brier score of 0.163. When limited to tumours with a diameter of ≤ 3 cm, the decision curve showed validity with a predictive probability threshold of 7–82% for highly invasive lung cancer and 18–93% for the control group. When limited to tumours with a diameter of ≤ 2 cm, the decision curve showed validity with a predictive probability threshold of 4–79% for highly invasive lung cancer and 21–93% for the control group.

### The comparison with CT findings

The results of ENS were compared with CTR which is current diagnostic criteria. Since CTR is generally measured with thin-slice CT, thin-slice CT if taken were used to compare with ENS in all patients. Thin-slice CT was performed in 698 patients. Results are shown in Table 3, and ROC curves are shown in Supplementary Fig. 3. For all patients, the AUC of the CTR was 0.73, and with a cut-off value of 0.5, the accuracy was 0.712, sensitivity 0.991, and specificity 0.224, while with a cut-off value of 1.0, the accuracy was 0.726, sensitivity 0.811, and specificity 0.577. ENS achieved an AUC of 0.882, accuracy 0.811, sensitivity 0.888, and specificity 0.675. For the 488 cStage IA patients, the AUC of the CTR was 0.701, and with a cut-off value of 0.5, the accuracy was 0.570, sensitivity 0.982, and specificity 0.211, while with a cut-off value of 1.0, the accuracy was 0.648, sensitivity 0.727, and specificity 0.579. ENS had an AUC of 0.811, accuracy of 0.736, sensitivity of 0.780, and specificity of 0.697.

**Table 3:** ENS and CTR performance for all patients and cStage IA

	AUC	Accuracy	Sensitivity	Specificity
All cStage (*n* = 873)				
ENS	0.882	0.811	0.888	0.675
CTR (cutoff)	0.73			
0.500		0.712	0.991	0.224
1.000		0.726	0.811	0.577
cStage IA (*n* = 488)				
ENS	0.811	0.736	0.780	0.697
CTR (cutoff)	0.701			
0.500		0.570	0.982	0.211
1.000		0.648	0.727	0.579

Comparison of ENS and CTR. The AUC, accuracy, sensitivity, and specificity are shown for all cases or limited to cStage IA. CTR cut-offs of 0.5 and 1 were tested. *AUC*, area under the curve; *ENS*, ensemble; *CTR*, consolidation tumour ratio

## Discussion

To our knowledge, this is the first report on developing machine learning models for predicting highly invasive lung cancer using preoperative PET/CT. A machine learning model for predicting pathological highly invasive lung cancer was established based on radiomics features extracted from PET/CT in a large cohort. The best predictive performance was achieved by combining PET and CT features and ensemble multiple machine learning models.

Seven machine learning models were applied in this study. Gradient boosting is generally shown to perform well on tabular data [27], but the excellent performance of deep models has been reported in recent years. Not only DNN with a few hidden layers [28] but also models designed for tabular data have been proposed [25]. When limited to AUC alone, ENS showed the best results, with an AUC of 0.856 (SD 0.0.183) for CT, AUC of 0.872 (SD 0.0177) for PET, and AUC of 0.880 (SD 0.0165) for PET/CT. Ensemble with unweighted simple averaging necessarily show superior results, especially when the models are of similarly high performance and low correlation [29]. All models in this study had excellent prediction performance, and especially DNN had low correlation coefficients with the other models. When combining multiple models, selecting a deep model with a low correlation coefficient may lead to better results. On the other hand, LR showed high prediction performance in PET/CT alongside ENS, indicating that classical models could be effective even with high-throughput omics data. When the risk of comparison bias was low, LR was indicated to show no marked difference in AUC from other machine learning models [30], so it is important to construct and combine multiple machine learning models for each analysis.

Lobectomy has long been the standard procedure for managing lung cancer. In 1995, a randomised trial compared lobectomy and sublobar resection for lung cancers with a diameter ≤ 3 cm. The results showed that sublobar resection was associated with a threefold risk of local recurrence and an increased mortality rate [31]. However, recently, small lung cancers with a high rate of ground-glass opacity found on thin-slice CT were shown to be primarily pathological non-highly invasive lung cancers [11]. In surgery, lung cancer with a low CTR showed a good recurrence-free survival, even with sublobar resection [32]. For patients whose cancer was inoperable due to their age or presence of major comorbidities, stereotactic body radiotherapy (SBRT) has also been shown to be effective and to have a low recurrence rate in cases of lung cancer with a low CTR, similar to surgery [33, 34]. However, as reported in JCOG0802/WJOG4607L, although sublobar resection was superior to lobectomy in terms of the overall survival, local recurrence was increased in the patients with a high CTR. SBRT has also been shown to increase local recurrence when the CTR is high [34]. Regarding surgery, the risk of local recurrence increases with proximity to the surgical margins [35, 36]. As for tumour effects, local recurrence is more common in highly invasive lung cancer than the other lung cancer [8, 37], and even in Adc, the presence of micropapillary patterns [38] or spread through air space (STAS) [39] is associated with an increased risk of recurrence. However, because the evaluation of invasiveness requires assessing extant invasion into anatomic structures, it is difficult to practically diagnose pathological highly invasive lung cancer preoperatively in cases without lymph node metastasis. CT effectively predicts invasiveness, provided that the findings reflect the pathologic invasion findings [11]. As shown by Aokage et al. [10], although there is a strong correlation between CT findings and invasiveness, CT findings do not always match the pathologic invasive findings in individual cases.

Several reports have shown that PET findings also effectively predict pathological highly invasive lung cancer. Li et al. showed that the MTV obtained from preoperative PET/CT was an independent predictor of lymphatic invasion, with an AUC of 0.854 when multiple factors were combined in the same cohort (not validation data) [40]. Despite the good statistical performance, however, no machine learning model has been built to predict highly invasive lung cancer based on PET/CT findings. Regarding machine learning models in lung cancer, Zhou et al. analysed PET images using the gradient boosting decision tree (GBDT) as a feature selector and classifier to differentiate between primary lung cancer and metastatic lung tumours, achieving an AUC of 0.983 [41]. In the same study, the GBDT was similarly used as a feature selector and classifier to differentiate lung Adc from lung Sqc, achieving an AUC of 0.839. Zhang et al. predicted the presence of an EGFR mutation on pretreatment PET/CT in non-small-cell lung cancer [42]. The least absolute shrinkage and selection operator (LASSO) algorithm was used to achieve an AUC of 0.87. A nomogram including the radiomics signature score by LASSO was also created within the same study, and the calibration plot showed consistency. Both studies had high predictive performance, and these findings along with the good results in the present study support the potential utility of machine learning models based on PET/CT images.

In our study, the ENS model was superior to the CTR both for all cases and when restricted to cStage IA cases. The accuracy was 9.9% higher for all patients and 16.6% higher for cStage IA patients than for those with a CTR cut-off of 0.5. When the cut-off was set at 1.0, the accuracy was 8.5% higher for all patients and 8.8% higher for cStage IA. The CTR tends to be prone to producing false positives, and machine learning models based on PET/CT, which show good overall performance, are valid. However, simply predicting whether or not a patient has highly invasive lung cancer is not sufficient for clinical use. While lobectomy is the standard resection approach, an essential aspect of performing sublobar resection involves minimizing false-negative results of highly invasive lung cancer. However, when a patient undergoes passive sublobar resection due to respiratory deterioration or a comorbidity, some risk of recurrence is assumed to be acceptable when the risk–benefit balance is considered.

It is important to note that the predicted probability of highly invasive lung cancer and the actual percentage of highly invasive lung cancer should approximate each other. Each patient has a different threshold for the predicted probability of highly invasive lung cancer in surgical selection. The treatment plan should be based on the individual risk–benefit balance, and a DCA that provides quantitative value criteria would be beneficial. The present study presented calibration plots and the results of a DCA for highly invasive and less-highly invasive lung cancer. The calibration plot approximated the actual percentage of highly invasive lung cancer when all cases were analysed and when cases were limited. The DCA indicated that the model was valuable over a wide range of threshold values. If the predicted probability of pathological highly invasive lung cancer could be presented for each patient, it might be useful for a more detailed consideration of therapeutic strategy.

Several limitations associated with the present study warrant mention. First, this study was a single-centre retrospective study, and the influence of bias cannot be excluded. Especially in the PET-CT analysis, it is technically complicated to analyse multiple scanners because the reference value differs for each scanner. Therefore, we limited our analysis to a single scanner. Conversely, whether or not the same level of prediction performance can be achieved for other PET images is unclear. Prediction models should be conducted for PET images of multiple scanners at multiple facilities using harmonization in the future to generalise these results. Second, this study was conducted with sublobar resection in mind. Although an increased risk of recurrence has been shown for highly invasive lung cancer, the comparison should be made with actual recurrence and overall survival rates. This study only concerns the prediction of highly invasive lung cancer and does not analyse the risk of recurrence or survival. Whether or not these models are clinically useful needs to be determined based on the postoperative course, so further analyses are warranted. A multicentre prospective analysis of machine learning model applications for the discrimination of invasiveness in preoperative lung cancer might be able to resolve this issue. Third, segmentation was performed using a method that eliminated manual segmentation as much as possible, but complete automatic segmentation was not achieved. To ensure reproducibility, it will be necessary to perform analyses based on a uniform procedure or automatic segmentation.

In conclusion, the machine learning models based on preoperative PET/CT findings accurately predicted pathological highly invasive lung cancer in the present study. In ENS, the accuracy was 81.1% for all cases and 73.6% for cStage IA, showing an improvement in accuracy of more than 8% over the CTR. This model predicts with high accuracy invasiveness that cannot be evaluated by CT alone, so it may be useful for determining treatment indication. The predicted probability and actual percentage of pathological highly invasive lung cancer were well approximated, and a DCA indicated that the models could provide validity with a wide range of thresholds in clinical analyses. Machine learning models of FDG-PET findings provided accurate information for predicting highly invasive lung cancer and may aid in the selection of surgical procedures.

## Supplementary Information

Below is the link to the electronic supplementary material.

Supplementary file1 (PDF 3489 kb)
Supplementary Figure 1. Schematic diagram of the study. (A) The process of feature extraction from CT and PET images, standardization and feature selection is shown. Combined CT and PET features are created by merging CT and PET features. (B) The patient cohort was divided 70% into a training set and 30% into a test set. The hyperparameter was tuned by the five-fold cross-validation method. The mean value and standard deviation of evaluation metrics were calculated after 100 iterations. (C) ENS calculates the predictive probability of pathological highly invasive cancer in all cases. The obtained results were used as the basis for evaluations

Supplementary file2 (PDF 421 kb)
Supplementary Figure 2. ROC curves for select cases are shown. (A) Adenocarcinoma, (B) squamous cell carcinoma, (C) tumors with a diameter ≤3 cm, and (D) tumors with a diameter ≤2 cm

Supplementary file3 (PDF 411 kb)
Supplementary Figure 3. ROC curves for the ENS model and consolidation tumor ratio. The blue line is based on the ENS model and the black line on CTR. (A) ROC curves for all cases. (B) ROC curves restricted to cStage IA

Supplementary file4 (DOCX 16 kb)

Supplementary file5 (DOCX 19 kb)
