# Supplementary material for: Predicting pathological highly invasive lung cancer from preoperative [18F]FDG PET/CT with multiple machine learning models
Source: Eur J Nucl Med Mol Imaging. 2022 Nov 17;50(3):715–26. doi: 10.1007/s00259-022-06038-7 (PMC9852187; doi:10.1007/s00259-022-06038-7)
Supplement: Supplementary file 1 — Supplementary file1 (PDF 3489 kb) Supplementary Figure 1. Schematic diagram of the study. (A) The process of feature extraction from CT and PET images, standardization and feature selection is shown. Combined CT and PET features are created by merging CT and PET features. (B) The patient cohort was divided 70% into a training set and 30% into a test set. The hyperparameter was tuned by the five-fold cross-validation method. The mean value and standard deviation of evaluation metrics were calculated after 100 iterations. (C) ENS calculates the predictive probability of pathological highly invasive cancer in all cases. The obtained results were used as the basis for evaluations [file 259_2022_6038_MOESM1_ESM.pdf]

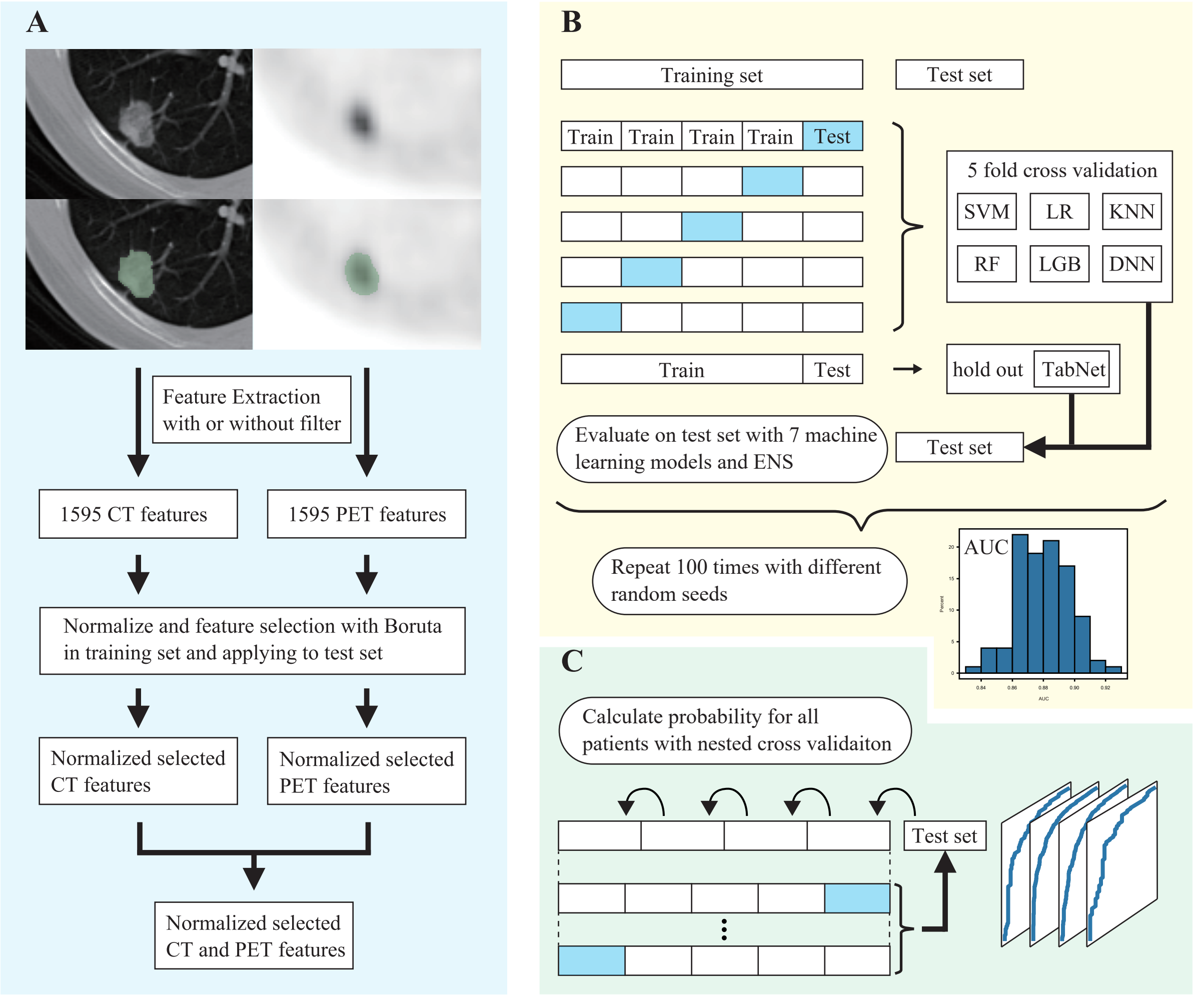
A
Feature Extraction with or without filter
1595 CT features
1595 PET features
Normalize and feature selection with Boruta in training set and applying to test set
Normalized selected CT features
Normalized selected PET features
Normalized selected CT and PET features
B
Training set
Test set
Train
Train
Train
Train
Test
5 fold cross validation
SVM
LR
KNN
RF
LGB
DNN
Train
Test
hold out
TabNet
Evaluate on test set with 7 machine learning models and ENS
Test set
Repeat 100 times with different random seeds
AUC
C
Calculate probability for all patients with nested cross validaiton
Test set
