# Supplementary figures and images for: Predicting pathological highly invasive lung cancer from preoperative [18F]FDG PET/CT with multiple machine learning models
Source: Eur J Nucl Med Mol Imaging. 2022 Nov 17;50(3):715–26. doi: 10.1007/s00259-022-06038-7 (PMC9852187; doi:10.1007/s00259-022-06038-7)

A

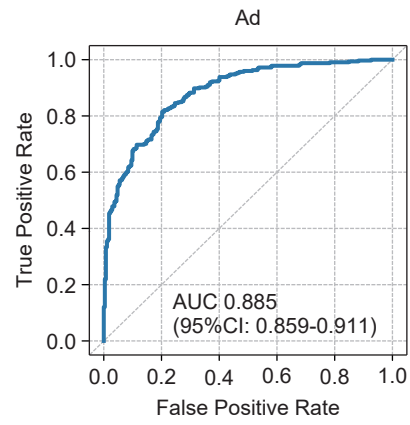

B

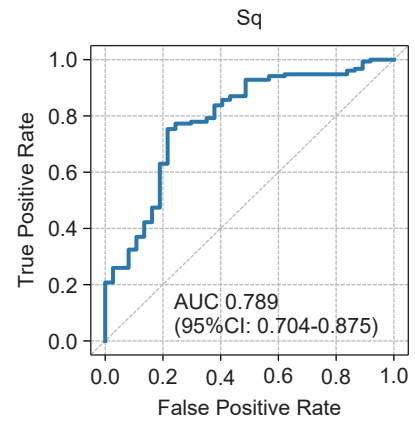

C

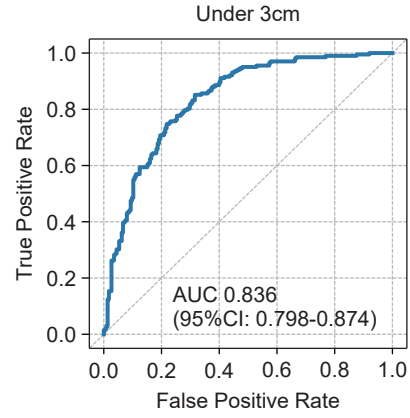

D

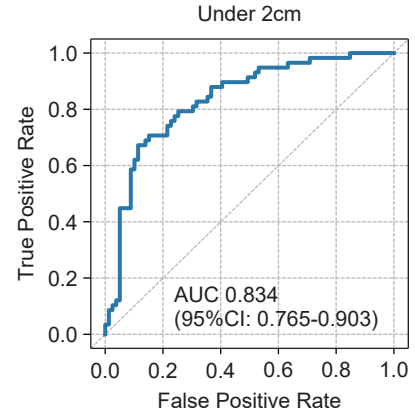

Supplement: Supplementary file 2 — Supplementary file2 (PDF 421 kb) Supplementary Figure 2. ROC curves for select cases are shown. (A) Adenocarcinoma, (B) squamous cell carcinoma, (C) tumors with a diameter ≤3 cm, and (D) tumors with a diameter ≤2 cm [file 259_2022_6038_MOESM2_ESM.pdf]

A

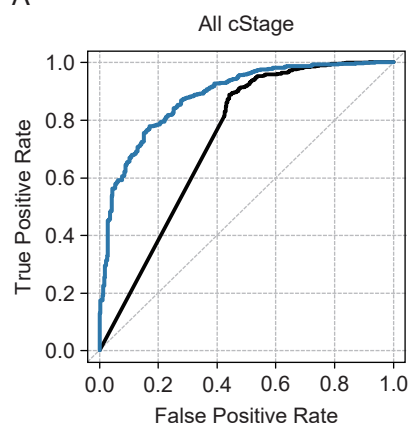

B

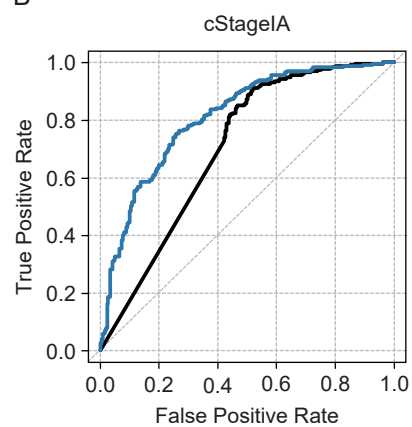

Supplement: Supplementary file 3 — Supplementary file3 (PDF 411 kb) Supplementary Figure 3. ROC curves for the ENS model and consolidation tumor ratio. The blue line is based on the ENS model and the black line on CTR. (A) ROC curves for all cases. (B) ROC curves restricted to cStage IA [file 259_2022_6038_MOESM3_ESM.pdf]
