# Supplementary material for: Predicting pathological highly invasive lung cancer from preoperative [18F]FDG PET/CT with multiple machine learning models
Source: Eur J Nucl Med Mol Imaging. 2022 Nov 17;50(3):715–26. doi: 10.1007/s00259-022-06038-7 (PMC9852187; doi:10.1007/s00259-022-06038-7)
Supplement: Supplementary file 4 — Supplementary file4 (DOCX 16 kb) [file 259_2022_6038_MOESM4_ESM.docx]

**Supplementary table1. Tuned hyperparameters in the machine learning models**

|  | Hyperparameters |
| --- | --- |
| LR | - C: {0.0001, 0.001, 0.01, 0.1, 1, 10, 100, 1000} - max_iter: {1000} |
| SVM | - C: {0.0001, 0.001, 0.01, 0.1, 1, 10, 100, 1000} - kernel: {rbf} - gamma: {0.001, 0.01, 0.1, 1, 10} |
| KNN | - n_neighbors: {1, 2, 3, 4, 5, 6, 7, 8, 9, 10, 15, 20} - weights: {uniform, distance} |
| RF | - max_depth: {3, 4, 5, 6, 7} - n_estimators: {1000} - max_features: {4, 5, 6, 7, 8} |
| LGB | - objective: {binary} - max_depth: {-1} - n_estimators: {1000} - num_leaves: {2, 3, 4, 5, 6, 7, 8, 13, 22, 31} - min_data_in_leaf: {1, 10, 20} - learning_rate: {0.005} - bagging_fraction: {0.8} - feature_fraction: {0.8} - bagging_freq: {1, 5} - max_bin: {1000} |
| DNN | - Number of layers: {4} - learning rate (lr): {0.001, 0.003, 0.005, 0.01} - max_epochs: {10} - iterator_train__batch_size: {16, 32, 64} |
| Tabnet | Pretrainer parameters   - n_d: {16} - n_a: {16} - n_steps: {2} - gamma: {1.7} - lambda_sparse: {0.001} - scheduler_params: patience: {5}, min_lr: {0.00005}, factor: {0.9}   TabNetClassifier   - max_epochs: {5000} - patience: {100} - batch_size: {16} - virtual_batch_size: {8} |

*LR*, Logistic Regression; *SVM*, Support Vector Machine; *KNN*, K-Nearest Neighbour; *RF*, Random Forest; *LGB*, Light Gradient Boosting Machine; *DNN*, Deep neural net; *ENS*, Ensamble
