# Supplementary material for: Predicting pathological highly invasive lung cancer from preoperative [18F]FDG PET/CT with multiple machine learning models
Source: Eur J Nucl Med Mol Imaging. 2022 Nov 17;50(3):715–26. doi: 10.1007/s00259-022-06038-7 (PMC9852187; doi:10.1007/s00259-022-06038-7)
Supplement: Supplementary file 5 — Supplementary file5 (DOCX 19 kb) [file 259_2022_6038_MOESM5_ESM.docx]

**Supplementary Table 2. Performance of the machine learning models for the training set**

|  | CT_AUC (SD) | PET_AUC (SD) | CT and PET_AUC (SD) |
| --- | --- | --- | --- |
| LR | 0.863 (0.00932) | 0.881 (0.00761) | 0.889 (0.00748) |
| SVM | 0.8552 (0.00928) | 0.878 (0.00791) | 0.881 (0.00737) |
| KNN | 0.855 (0.0114) | 0.872 (0.00973) | 0.881 (0.00858) |
| RF | 0.863 (0.00907) | 0.883 (0.00888) | 0.889 (0.00760) |
| LGB | 0.865 (0.00969) | 0.885 (0.00921) | 0.894 (0.00838) |
| DNN | 0.865 (0.00982) | 0.882 (0.00751) | 0.890 (0.00732) |
| Tabnet | 0.872 (0.0305) | 0.885 (0.0297) | 0.896 (0.0284) |

*CT*, computed tomography; *AUC*, area under the curve; *SD*, standard division; PET, positron emission tomography; *LR*, Logistic Regression; *SVM*, Support Vector Machine; *KNN*, K-Nearest Neighbour; *RF*, Random Forest; *LGB*, Light Gradient Boosting Machine; *DNN*, Deep neural net; *ENS*, Ensamble
